# Cardioprotective effects of dietary lipids evident in the time‐dependent alterations of cardiac function and gene expression following myocardial infarction

**DOI:** 10.14814/phy2.12019

**Published:** 2014-05-20

**Authors:** Jessica M. Berthiaume, Salaman M. Azam, Brian D. Hoit, Margaret P. Chandler

**Affiliations:** 1 Department of Physiology and Biophysics, Case Western Reserve University, Cleveland, Ohio; 2 Department of Medicine, Case Western Reserve University and University Hospitals Case Medical Center, Cleveland, Ohio

**Keywords:** AKT, energy metabolism, gene expression, high dietary fat, left ventricular dysfunction, myocardial infarction, pyruvate dehydrogenase, PI3K

## Abstract

We have previously shown that prolonged high–saturated fat feeding (SAT) for 8 weeks after myocardial infarction (MI) improves ventricular function and prevents the metabolic remodeling commonly observed in heart failure. The current study was designed to delineate the interplay between markers of energy metabolism and indices of cardiac remodeling with 2 and 4 weeks of post‐MI SAT in male Wistar rats. By 2 weeks, less remodeling was noted in MI‐SAT evidenced by diminished chamber dilation and greater ejection fraction assessed by echocardiography and hemodynamic measures. In addition, gene expression of energy metabolism targets involved in FA uptake, oxidation, and glucose oxidation regulation was increased in MI‐SAT with respect to MI alone, although no change in PDH phosphorylation was observed. The regulatory kinase, phosphoinositide 3 kinase (*Pi3k*), was strongly induced by 2 weeks in the MI‐SAT group, although AKT protein content (a primary downstream target of PI3K that affects metabolism) was decreased by both MI and SAT alone, indicating early involvement of cellular signaling pathways in lipid‐mediated cardioprotection. Our results demonstrate that cardioprotection occurs acutely with SAT following MI, with improvement in indices of both cardiac function and fatty acid oxidation, suggesting a mechanistic role for energy metabolism in the beneficial effects of high dietary fat following cardiac injury.

## Abstract (precis)

e12019

A diet rich in saturated fats is cardioprotective after myocardial infarction. The cardioprotective effect is noted by 2 weeks and includes functional and genomic changes indicative of a relationship with preservation of metabolic flexibility.

## Introduction

Myocardial infarction (MI) is a significant healthcare concern and annual incidence rates have steadily increased in many postindustrial countries (Go et al. 2013). Lipids have classically been regarded as detrimental to cardiovascular health because high circulating concentrations are correlated with the risk of developing cardiovascular disease and heart failure (HF) (Djousse et al. 2013). However, the association of lipids with adverse effects in cardiovascular health has more recently been recognized to be altered in the context of cardiac injury. The occurrence of cardiac events and cardiac‐related death is actually lower in HF patient populations consuming a greater percentage of their caloric intake as fat (Mozaffarian 2005; Tuttle et al. 2008). This would suggest that the role of lipids in the injured myocardium impacts different cellular pathways and/or processes than in the normal myocardium and this potentially reflects the dynamic metabolic adaptation which occurs in the compromised heart.

Within cardiomyocytes, lipids participate in a number of biological processes, yet how they impact pathology in the heart remains to be fully elucidated. A prominent role for fatty acids (FAs) in the cardiomyocyte is as a fuel for energy metabolism as a constant supply of ATP is required to sustain pump action (Saddik and Lopaschuk 1991). Studies on end‐stage HF patients have demonstrated an increased reliance on glucose over FAs accompanied by an overall depression in ATP production implying cardiac function is intimately related to the capacity for energy metabolism (Conway et al. 1991; Sack et al. 1996; Beer et al. 2002; Tuunanen et al. 2006; Ingwall 2009). Considerable research efforts have sought to characterize how metabolic substrate selection impacts the onset and progression of HF. Historically, management of acute cardiac injury through metabolic manipulations has focused on promoting glucose utilization and suppressing fatty acid oxidation (FAO), although this approach is now recognized to have limited success in the long‐term prognosis of HF (Diaz et al. 2007). Studies targeting energy metabolism in the injured heart both by pharmacological enhancement of glucose utilization and FAO inhibition have demonstrated improvements in cardiac function, although many of the drugs employed have additional cellular targets that may underlie their mechanism of action (Ardehali et al. 2012).

A number of studies, including our own, have demonstrated that increased dietary lipid following cardiac injury positively impacts function and metabolism (Okere et al. 2005; Rennison et al. 2007; Chess et al. 2008). We have shown that 8 weeks of a high–saturated fat diet (SAT) post‐MI in a rat model does not exacerbate dysfunction and, in fact, cardiac function and indices of fatty acid metabolism are improved compared to normal chow pair‐fed rats (Rennison et al. 2007; Berthiaume et al. 2010; Christopher et al. 2010). This cardioprotection is apparent without alterations to scar size, suggesting improved performance of the viable myocardium through increase in the utilization of FAs for ATP production to positively impact cardiac function. As the development and progression of cardiac dysfunction following infarction is dynamic over time, the point at which high fat confers improvements to the myocardium is critical to understand how lipids impact the heart. On the basis of our previous findings, we hypothesize that the cardioprotective effect of high dietary fat is established very early and that the relationship between contractile function and indices of cardiac metabolism at these earlier time points will provide insight into the underlying mechanism(s) of protection and related cellular processes. Specifically, we aimed to define function and expression profiles at 2 and 4 weeks following MI with high‐fat feeding to gain additional insight into the primary mechanism of dietary lipid‐mediated cardioprotection. Defining a shorter period of intervention may also inform potential therapeutic approaches related to metabolic modulation after cardiac injury.

## Materials and Methods

### Reagents

All chemicals were of research grade or higher and were purchased from ThermoFisher, Pittsburgh, PA or Sigma Aldrich, St. Louis, MO unless noted otherwise.

### Animal treatment and surgical procedures

**Figure 1.**
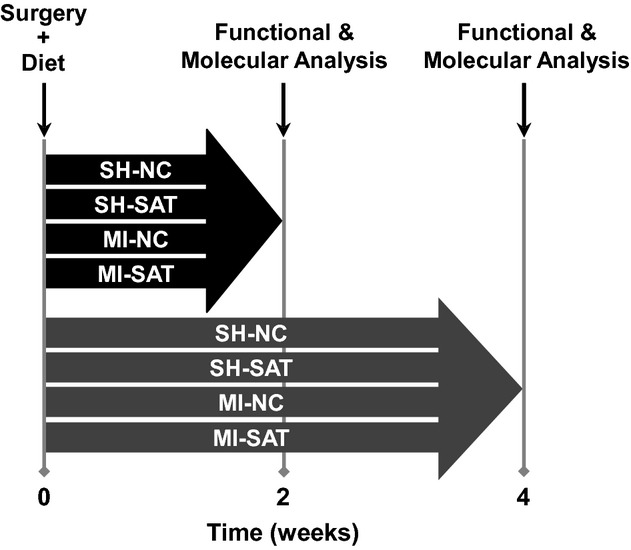
Diagram depicting study design. Surgical procedures to induce myocardial infarction (MI) were followed by access to diets on day 0. Rats were randomly assigned to surgical and dietary groups, then to either functional assessment cohort (echocardiography or in vivo hemodynamics). One cohort of rats was assessed at 2 weeks, the other at 4 weeks. *LV tissue and plasma was sampled for biochemical and gene expression analysis at the termination of experiment. SH, sham; MI, myocardial infarction; NC, normal chow; SAT, high–saturated fat chow (see text for additional technical details).

All animal procedures were approved by the Case Western Reserve University Institutional Animal Care and Use Committee (IACUC). Adult, male Wistar rats (200–250 g) were maintained on a reverse light/dark cycle and all surgical procedures were performed during the dark cycle to standardize circadian effects on metabolism and cardiovascular function (Stavinoha et al. 2004). Figure 1 depicts the experimental design for this study in which two groups were generated in parallel for each time point and assessed for function by the methodologies described below. Rats were randomly assigned to a surgical group and underwent either a coronary ligation surgery to induce myocardial infarction (MI) or a sham (SH) surgery in which the suture was not tied, but merely looped through the myocardium (Morgan et al. 2004). Infarctions were visually confirmed by blanching of the affected myocardium prior to chest closure. Mortality with this procedure was approximately 30% and typically occurred prior to the end of the recovery period after the surgery, consistent with previous work (Rennison et al. 2007). Rats were allowed to recover, then randomly allowed access to a normal (NC in contributions of kcal/g; 60% carbohydrate, 26% protein, and 14% kcal fat, LabDiet Prolab Isopro RMH 3000, St Louis, MO) or high saturated fat (SAT; 20% kcal carbohydrate, 20% protein, and 60% fat; 33:25:33% stearate:palmitate:oleic acid, Research Diets, D04051705; New Brunswick, NJ) chow; the caloric intake between these diets is similar as previously characterized (Okere et al. 2006). Rats were maintained on the diets for 2 or 4 weeks after which cardiac function was assessed by either echocardiography (*n *=**6–8 per group) or in vivo hemodynamic assessment of left ventricular (LV) function (*n *=**6–12 per group) in the fed state. Rats were euthanized and hearts were quickly excised; the scar was carefully trimmed from the LV and weighed. The remaining LV tissue and a plasma sample was frozen in liquid nitrogen and stored at −80°C for future analysis.

### Echocardiography and strain analysis

After 2 or 4 weeks, rats were anesthetized and echocardiography was performed to assess LV function as described previously (Morgan et al. 2004). Two‐dimensional (2D) short‐ and long‐axis‐directed M‐mode, and 2D recordings were acquired with a 15 MHz transducer using a Siemens Sequoia C256 system (Siemens Medical Solutions, Mountain View, CA) by a blinded technician. End‐diastolic and ‐systolic areas were measured and relative wall thickness (RWT, defined by: [anterior + posterior wall thickness at diastole]/end‐diastolic diameter), area fractional shortening and ejection fraction were calculated. Rats in the MI groups were excluded from analysis if they did not meet the minimum criteria for LV dysfunction of an ejection fraction (EF) <70% and fractional shortening (FS) <0.40.

Acoustic capture B‐mode cine clips (120 Hz) were acquired using electrocardiographic gating in the Siemens Sequoia System as previously described (Azam et al. 2012). Briefly, image processing and data analysis was done offline with Syngo^™^ Vector Velocity Imaging technology software (Siemens Medical Solutions, Mountain View, CA). B‐mode clips were selected based on satisfactory visualization of the endocardial border in the absence of image artifacts. Epi‐ and endocardial LV borders were traced manually; tracking accuracy was verified over three cycles in the parasternal short‐axis view at end systole. Strain (Lagrangian) values were generated for the base, mid, and apex of the LV. Peak radial and circumferential radial strain values for each LV segment were then averaged to obtain global radial strain (GRS) and global circumferential strain (GCS) values for each heart. High‐quality images for 2D speckle‐tracking‐based strain analyses were obtained by performing image acquisition at a high frame rate by using the smallest possible depth and sector size. All image acquisitions and offline measurements were conducted by a single, blinded investigator (interobserver differences in circumferential and radial strains were −3.5 ± 14.7 and −3.9 ± 21.9 [mean ± SD], respectively; Azam et al. 2012).

### Hemodynamic evaluation of LV function

Following 2 or 4 weeks, rats were anesthetized with isoflurane (1.5–2.0%) and a pressure–volume transducer (2.0 Fr; Millar instruments, Houston, TX) was introduced into the LV via the right carotid artery and signals were recorded as described in previously published methods (Pacher et al. 2008; Berthiaume et al. 2010). Pressure and volume signals were recorded through a MPVS 300 system (Millar Instruments) with output to LabChart^®^ data acquisition software (AD Instruments, Colorado Springs, CO). End‐diastolic volume (EDV) and stroke volume (SV) values obtained from echocardiography were used to calibrate volume data through the point and difference function available in LabChart^®^. Hemodynamic parameters such as heart rate, ventricular pressures and volumes, stroke work, and ±d*P*/d*t* were analyzed using PVAN software (AD Instruments).

### Plasma and tissue metabolite analysis

Left ventricular tissue and plasma samples were assayed to quantify triglyceride content. Triglycerides (TG) were extracted from 50 mg of frozen LV tissue homogenized in four volumes of 2:1 (v:v) chloroform:methanol. Homogenates were centrifuged at 2000*g* for 15 min and supernatants were evaporated to dryness in a chemical hood. Dried residues were resuspended in assay reagents and quantitated using an enzyme‐coupled spectrophotometric assay along with a standard curve according to manufacturer's instructions (Type TG‐M and TG‐H kits; Wako Chemicals, Osaka, Japan). For plasma analysis, 20 *μ*L was added directly to the assay.

### Targeted gene expression profiling by quantitative PCR

Left ventricular tissue was powdered in liquid nitrogen and used for total RNA isolation. Samples were homogenized using the TissueLyser system, processed as suggested by the manufacturer (Qiagen, Valencia, MD). Total RNA was extracted with standard phenol:chloroform methods with isopropanol precipitation subsequent to homogenization of 50 mg powdered tissue in 1.0 mL of QIAzol^™^ lysis reagent (Qiagen). The resulting RNA pellet was resuspended in 100 *μ*L of molecular biology grade water and further purified by an on‐column clean up with DNase digestion according to manufacturer's suggestions (RNeasy MinElute Cleanup Kit; Qiagen). RNA was quantitated and assessed for purity using a NanoDrop spectrophotometer (ThermoFisher). Reverse transcription to generate cDNA was performed with 1.0 *μ*g of total RNA using the iScript^™^ cDNA Synthesis Kit according to manufacturer's instructions (Bio‐Rad, Hercules, CA) and a mixture of oligo‐dT:random primers (80:20 molar ratio). Primers were designed using an online version of Primer3 available through NCBI (http://www.ncbi.nlm.nih.gov/tools/primer-blast/; Waldo et al. 2013). Sequence and gene identifier information is presented in Table 1 for each target; 18s rRNA was used for target normalization. Reaction efficiency of primer sets was optimized by comparing standard curve slope fits at various primer concentrations. Primer specificity was confirmed by melting curve analysis and gel electrophoresis to verify a single product peak at the predicted melting temperature and a single band at the correct molecular weight, respectively. Gene expression was evaluated by qPCR using SYBR green detection with a prepared mastermix according to manufacturer's suggestions (PerfeCta^™^ Supermix; Quanta Biosciences, Gaithersburg, MD). Reactions were run in 96‐well plates in the StepOnePlus system (Applied Biosystems, Grand Island, NY). PCR data are presented as relative expression values corresponding to the normalized, log‐transformed threshold cycle of amplification (*C*_t_) as defined in previously published calculations (Schmittgen and Livak 2008). In addition to the 2 and 4 week samples used for qPCR analysis, archived tissue samples from rats undergoing the same procedure, but allowed 8 weeks of post‐MI SAT feeding were also processed to generate comparative data which are presented in Figure 4.

**Table 1.** Gene and primer sequence information for qPCR evaluation of gene expression.

Gene	Gene Symbol	Accession ID	Forward primer 5′→3′	Reverse primer 5′→3′	Product size (base pairs)
18s rRNA	*18s*	NR_046237.1	cggtacagtgaaactgcgaat	gctgaccgggttggttttg	177
Acyl‐CoA dehydrogenase (medium chain)	*Acadm*	NM_016986.2	gagccgggactagggtttag	aagaccaccacaactctccg	201
Acyl‐CoA thioesterase 1	*Acot1*	NM_031315.1	accctgaggtaaaaggacca	ttgcaaagcatctacaacatcc	239
Angiopoietin‐like 4	*Angptl4*	NM_199115.2	gggacagacccgaaggatag	ggtcaagaggtcaatctggc	189
Atrial natriuretic peptide	*Nppa*	NM_012612.2	gagccgagacagcaaacatc	gtggtctagcaggttcttgaaat	180
Myosin heavy chain, *α*	*Myh6*	NM_017239.2	ctcacctaccagacagagga	acaggttattcctcatcgtgc	272
Myosin heavy chain, *β*	*Myh7*	NM_017240.1	agtcatggcggatcgagaga	cagtcaccgtcttgccattct	215
Phosphoinositide‐3‐kinase	*Pi3k*	NM_053923.1	gacacacagcaacctgtacc	tctctcatggtgcgagtctg	184
Pyruvate dehydrogenase kinase 4	*Pdk4*	NM_053551.1	ctgctgttcggttcaga	ctgggctcttttcgtggaac	205

### Western blotting

Frozen LV tissue was powdered and added to isolation buffer containing 20 mmol/L Tris (pH 7.8), 137 mmol/L NaCl, 2.7 mmol/L KCl, 1 mmol/L MgCl_2_, 1% (w/v) Triton X‐100, 10% (w/v) glycerol, 1 mmol/L EDTA, with protease (#78425, ThermoFisher) and phosphatase inhibitor cocktails (#5726 and #P0044; Sigma Aldrich) added. Tissue was homogenized followed by incubation at 4°C for 20 min and then centrifugation at 5000*g* for 10 min (4°C). Protein concentration was determined in the supernatant using a BCA protein assay kit (Pierce, Rockford, IL). Protein (10 *μ*g in Laemmli buffer) was run on a 4–20% SDS‐polyacrylamide gel (Bio‐Rad) and transferred to polyvinylidene difluoride (PVDF) (Bio‐Rad). Membranes were blocked with 5% BSA in Tris‐buffered saline plus Tween 20 (0.01% v:v), then incubated overnight with primary antibody. Antibodies: pyruvate dehydrogenase (PDH), 1:2000 dilution (Cell Signaling, Beverly, MA), phospho‐PDH Ser^293^ (pPDH), 1:2000 (Calbiochem, Gibbstown, NJ), PDH kinase 4 (PDK4), 1:2000 (gift from R. Harris) protein kinase B (AKT, aka PKB), 1:1000 (Cell Signaling), pAKT Ser^473^ and HSC70 (Santa Cruz, Paso Robles, CA) used as a loading control (1:5000). Following incubation with secondary antibodies (1:15000) chemiluminescent detection (Supersignal West Pico, Pierce, IL) was performed on standard autoradiography film (Denville, NJ) which was scanned at 720 dpi (Epson P4490, transmittance mode). Densitometric values were acquired using Image J software in histogram analysis mode (NIH, MD). Protein ratios were derived from sequential probing of membranes after stripping with Restore^©^ Western blot stripping buffer (Pierce, IL). Samples were run on a single gel to minimize gel‐to‐gel signal variations.

### Statistical analysis

Statistical differences were determined by use of a two‐way ANOVA with a Bonferroni post hoc analysis, with the exception of scar weights between the MI groups which were evaluated by a two‐tailed *t*‐test. A *P*‐value <0.05 was used as the criterion for significance. Data are presented as mean ± standard error of the mean (SEM).

## Results

### Animal characteristics and tissue/plasma substrate analyses

No changes in body weight (BW) or heart weight (HW) were observed with respect to diet (NC or SAT) or surgery (SH or MI) at either 2 or 4 weeks post‐MI with high‐fat feeding (Table 2). Additionally, scar weights were not different between MI‐SAT and MI‐NC hearts at 2 or 4 weeks. Triglyceride (TG) content of the plasma and myocardial tissue was increased in both SAT groups (SH and MI) with respect to their dietary controls (NC) at 2 weeks; a similar trend was apparent at 4 weeks.

**Table 2.** Animal characteristics and triglyceride analyses.

	SH‐NC	SH‐SAT	MI‐NC	MI‐SAT
2 Weeks				
Body weight (g)	369 ± 9	391 ± 7	378 ± 5	378 ± 7
Heart weight (g)	0.77 ± 0.02	0.81 ± 0.02	0.78 ± 0.01	0.80 ± 0.01
H:B weight ratio	2.10 ± 0.04	2.07 ± 0.03	2.08 ± 0.03	2.12 ± 0.04
Scar (g)	–	–	0.177 ± 0.008	0.195 ± 0.015
Scar:H weight ratio	–	–	0.227 ± 0.010	0.245 ± 0.020
TG_lv_ (mg/g)	1.27 ± 0.26	1.92 ± 0.27*	1.05 ± 0.14	1.54 ± 0.11*
TG_plasma_ (mg/mL)	0.322 ± 0.123	0.486 ± 0.107*	0.181 ± 0.030	0.343 ± 0.056*
4 Weeks				
Body weight (g)	413 ± 7	434 ± 10*	423 ± 5	433 ± 8*
Heart weight. (g)	0.88 ± 0.02	0.89 ± 0.03	0.86 ± 0.02	0.86 ± 0.02
H:B weight ratio	2.13 ± 0.04	2.04 ± 0.04	2.03 ± 0.03	1.98 ± 0.04
Scar (g)	–	–	0.181 ± 0.013	0.202 ± 0.009
Scar:H weight ratio	–	–	0.214 ± 0.017	0.236 ± 0.010
TG_LV_ (mg/g)	2.02 ± 0.55	2.82 ± 0.90	1.30 ± 0.28	1.74 ± 0.41
TG_plasma_ (mg/mL)	0.353 ± 0.068	0.933 ± 0.191*	0.425 ± 0.046	0.453 ± 0.077*

Data presented as the mean ± SEM (animal data, *n* = 13–19; TG data, *n* = 3–8).

* *P* < 0.05 versus surgical control (SH).

* *P* < 0.05 versus dietary control (NC).

* A main effect (*P* < 0.05) for diet (NC vs. SAT).

### Cardiac function following MI with 2 or 4 weeks of high‐fat feeding

**Figure 2.**
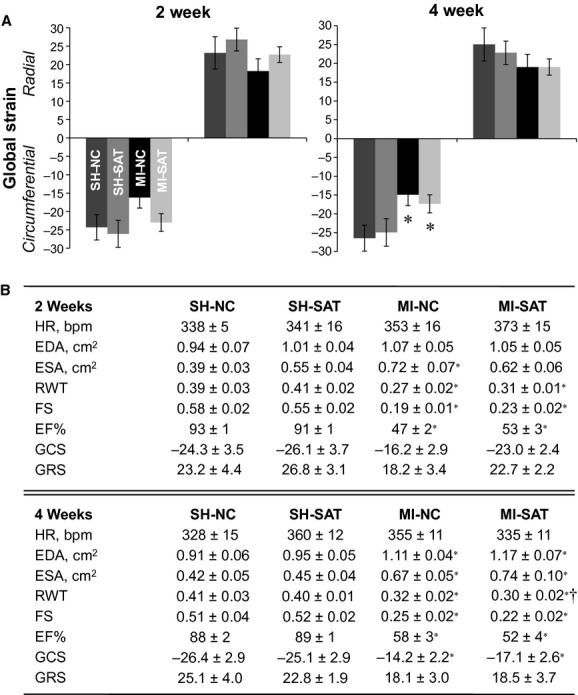
Cardiac function as measured by echocardiography and subsequent strain analysis in rats with 2 or 4 weeks of post‐MI high‐fat feeding. (A) Global strain values (circumferential, GCS; radial, GRS) generated from echocardiographic images using speckle‐tracking methodology (*n *=**6). (B) Echocardiographic parameters (*n *=**6–8). Values represent the mean ± standard error of the mean (SEM). *P <**0.05 versus surgical control (SH). ^†^*P *<**0.05 versus dietary control (NC).

Conventional and strain echocardiographic measures are shown in Figure 2. At 2 weeks, fractional shortening (FS), ejection fraction (EF), and relative wall thickness (RWT) were decreased in both MI groups. End‐diastolic area (EDA) was not altered at 2 weeks in the MI groups, whereas end‐systolic area (ESA) was significantly increased in the MI‐NC group. Global radial and circumferential strain (GRS and GCS, respectively) were not significantly altered in either MI group at the 2‐week time point, but trended toward improvement in the MI‐SAT animals. No differences in heart rate were observed for any of the groups at 2 weeks. At 4 weeks, FS, EF, and RWT remained depressed in the MI groups, however, RWT was modestly decreased in MI‐SAT compared to MI‐NC hearts. Additionally, the magnitude of GCS was diminished in the MI groups, with a trend of a smaller change in the MI‐SAT animals. No differences in heart rate were noted across groups at 4 weeks.

Invasive hemodynamic assessment was performed in a separate set of rats at 2 and 4 weeks (Table 3). As with the echocardiographic data, EF was decreased in both MI groups at both 2 and 4 weeks, but to a lesser extent in the MI‐SAT group at 2 weeks. No changes in end‐diastolic (LVEDP) or end‐systolic (LVESP) pressures were observed at either 2 or 4 weeks. LV end‐diastolic (LVEDV) and end‐systolic (LVESV) volumes were increased in both MI groups at 2 weeks, however, LV chamber dilation was diminished in the MI‐SAT group as compared to MI‐NC. Surprisingly, LVEDV was greater in MI‐SAT than the MI‐NC group in the 4 week cohort, however, this apparent increase in preload did not significantly impact ejection volumes as no difference between MI groups was found. Although stroke volume, stroke work, and cardiac output were depressed at 2 weeks in the MI groups, at 4 weeks the MI‐SAT group was improved as compared to MI‐NC. With respect to indices of contractility, +d*P*/d*t* and −d*P*/d*t* were reduced in the MI‐NC group at 2 weeks, although not in the MI‐SAT group. In contrast, +d*P*/d*t* and −d*P*/d*t* was decreased in both MI groups at 4 weeks, with a main effect for surgery. Arterial elastance was increased with respect to MI at 2 weeks, but was improved in MI‐SAT versus MI‐NC by 4 weeks.

**Table 3.** Hemodynamic parameters in the rat heart following MI with subsequent SAT feeding 2 and 4 weeks.

	SH‐NC	SH‐SAT	MI‐NC	MI‐SAT
2 Weeks				
Heart rate (HR), bpm	320 ± 16	357 ± 13*	317 ± 9	333 ± 9
LVESP, mmHg	102 ± 5	91 ± 7	98 ± 4	95 ± 4
LVEDP, mmHg	11 ± 1	12 ± 2	12 ± 1	13 ± 1
LVEDV, *μ*L	448 ± 6	453 ± 9	643 ± 4*	557 ± 6**
LVESV, *μ*L	89 ± 5	119 ± 14	415 ± 15*	300 ± 21**
Stroke volume, *μ*L	393 ± 4	375 ± 10	266 ± 7*	282 ± 21*
Stroke work, mmHg *μ*L 10^3^	34.17 ± 2.33	31.17 ± 1.69	20.89 ± 1.12*	22.04 ± 2.43*
Cardiac output mL/min	126 ± 6	134 ± 6	83 ± 2*	94 ± 7*
Ejection fraction, %	82 ± 1	78 ± 2	40 ± 1*	49 ± 4**
Elastance_ART_ mmHg/*μ*L	0.26 ± 0.01	0.24 ± 0.02	0.37 ± 0.02*	0.35 ± 0.02*
+d*P*/d*t*, mmHg/sec	7950 ± 563	7097 ± 359	6643 ± 318*	7097 ± 439
−d*P*/d*t*, mmHg/sec	−8329 ± 1014	−6486 ± 554*	−5668 ± 307*	−5596 ± 350
Max Power_PA_, mW/*μ*L^2^	5.65 ± 0.44	6.09 ± 0.68	1.82 ± 0.12*	2.63 ± 0.27*
4 Weeks				
Heart rate, bpm	330 ± 16	338 ± 13	321 ± 13	321 ± 16
LVESP, mmHg	95 ± 8	96 ± 4	105 ± 5	101 ± 4
LVEDP, mmHg	14 ± 3	13 ± 1	22 ± 4	19 ± 3
LVEDV, *μ*L	420 ± 23	461 ± 8*	586 ± 10*	687 ± 7**
LVESV, *μ*L	95 ± 12	103 ± 8	373 ± 31*	387 ± 24*
Stroke volume, *μ*L	359 ± 11	396 ± 10	284 ± 13*	359 ± 15**
Stroke work, mmHg *μ*L 10^3^	31.51 ± 1.63	37.46 ± 2.00*	21.75 ± 1.46*	28.55 ± 2.20**
Cardiac output mL/min	117.98 ± 3.91	133.78 ± 4.41	91.55 ± 6.27	115.25 ± 7.80**
Ejection fraction, %	81 ± 2	82 ± 2	47 ± 3*	50 ± 2*
Elastance_ART_ mmHg/*μ*L	0.27 ± 0.03	0.25 ± 0.01	0.37 ± 0.02*	0.28 ± 0.01*
+d*P*/d*t*, mmHg/sec	7248 ± 461	8221 ± 345	6518 ± 291*	6887 ± 324**
−d*P*/d*t*, mmHg/sec	−6768 ± 684	−8073 ± 420	−5757 ± 414*	−5702 ± 499**
Max Power_PA_, mW/*μ*L^2^	7.69 ± 1.80	6.27 ± 0.08	2.93 ± 0.31*	2.63 ± 0.28*

Data presented as the mean ± SEM (*n* = 6–12).

* *P* < 0.05 versus surgical control (SH).

* *P* < 0.05 versus dietary control (NC).

* A main effect (*P* < 0.05) for surgery (MI vs. SH).

### Expression changes in metabolic and stress genes with 2 and 4 weeks of high fat following MI

**Figure 3.**
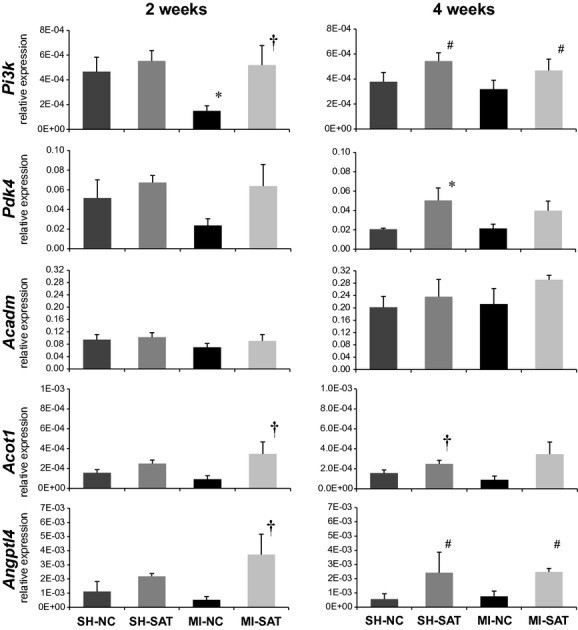
Gene expression changes of *Pi3k*,* Pdk4*,* Acadm*,* Acot1*, and *Angptl4* after 2 or 4 weeks of high‐fat feeding following MI. Quantitative PCR was performed to assess expression changes using the d*C*_t_ method and normalization to 18s rRNA. Values are the mean ± SEM (*n *=**4–5). *P <**0.05 versus surgical control (SH). ^†^*P *<**0.05 versus dietary control (NC). ^#^A main effect (*P *<**0.05) for diet (SAT vs. NC). Please refer to Table 1 for further information on selected genes and primer sets.

**Figure 4.**
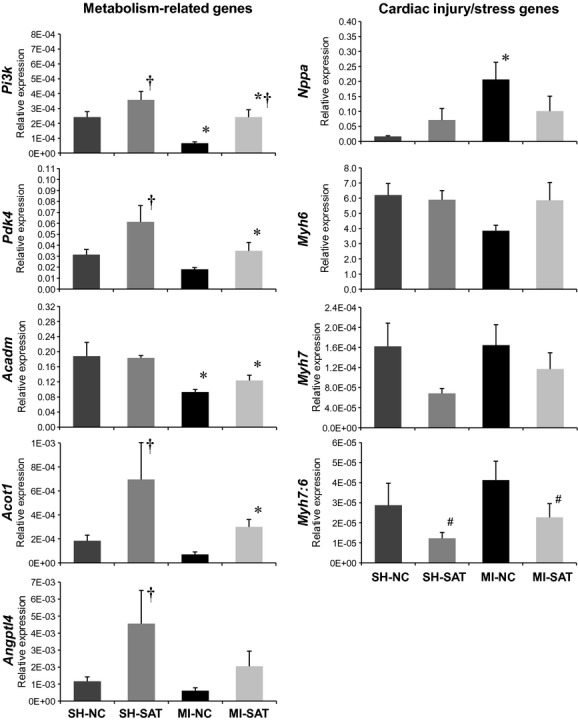
Gene expression changes of metabolism‐related genes (*Pi3k*,* Pdk4*,* Acadm*,* Acot1*, and *Angptl4*) and cardiac stress genes (*Nppa*,* Myh6*, and *Myh7*) at 8 weeks of high‐fat feeding following MI. Quantitative PCR was performed to assess expression changes using the d*C*_t_ method and normalization to 18s rRNA. Values are the mean ± SEM (*n *=**4–5). *P <**0.05 versus surgical control (SH). ^†^*P *<**0.05 versus dietary control (NC). ^#^A main effect (*P *<**0.05) for diet (SAT vs. NC). Table 1 contains details of the analyzed genes and primer sets.

To expand on our previous findings of gene expression changes between the MI groups (at 8 weeks using microarrays), we assessed a panel of genes at 2 and 4 weeks in all experimental groups and the results are shown in Figure 3. All gene targets were also evaluated in 8 week LV tissue to verify consistency with previous data from microarrays (Berthiaume et al. 2010) and allow comparison to 2 and 4 week data; the data are presented in Figure 4. In general, the expression of genes related to energy metabolism was depressed in the MI‐NC group; expression trends were similar across time and were consistent with our previously published data at 8 weeks (Rennison et al. 2008; Berthiaume et al. 2010). Phosphatidylinositide 3‐kinase (*Pi3k*), a kinase regulated by a number of receptors including the insulin receptor to regulate downstream metabolism targets, expression was significantly decreased at 2 weeks in the MI‐NC group compared to both the SH‐NC and MI‐SAT group, suggesting an early effect of injury. At 4 weeks, a main effect for diet (SAT vs. NC) was noted for the increase in *Pi3k* expression. At 8 weeks, *Pi3k* expression was downregulated in the MI‐NC group as compared to both SH‐NC and MI‐SAT, similar to the 2‐week profile (Figure 4). In addition, there was an increase in *Pi3k* gene expression in the SH‐SAT versus SH‐NC group, implying a diet‐induced impact. Pyruvate dehydrogenase kinase 4 (*Pdk4*), a kinase with inhibitory effect on PDH and inducible by high fat, expression in the MI‐NC group at 2 weeks trended downward compared to MI‐SAT (*P *=**0.053). At 4 weeks, *Pdk4* was significantly increased in the SH‐SAT group compared to SH‐NC. By 8 weeks, *Pdk4* expression was significantly increased in SH‐SAT versus SH‐NC group as noted at 2 weeks. Despite a trend of an increase, *Pdk4* in the MI‐SAT group was significantly decreased with respect to SH‐SAT. *Acadm*, an enzyme in the pathway of fatty acid oxidation, expression was not altered at either 2 or 4 weeks, but was significantly downregulated in both MI groups by 8 weeks. *Acot1*, an enzyme involved in intracellular FA handling, expression was significantly increased in MI‐SAT with respect to MI‐NC at 2 weeks and despite a similar trend, was not significantly altered at 4 weeks (*P *=**0.052). However, *Acot1* expression was upregulated at 4 weeks in the SH‐SAT group versus SH‐NC. By 8 weeks, *Acot1* expression was increased in the SH‐SAT compared to both the SH‐NC and MI‐SAT group. The expression of *Angptl4*, a secreted protein involved in a number of processes including regulation of FA uptake, was significantly increased at 2 weeks in the MI‐SAT group as compared to the MI‐NC group and at 4 weeks there was a main effect for diet. After 8 weeks, *Angptl4* expression in the SH‐SAT group was greater with respect to SH‐NC. In addition to metabolic targets, several genes commonly used as indictors of cardiac injury and remodeling were also investigated.

Circulating levels of natriuretic peptide A (gene: *Nppa*) are routinely measured as a biomarker of cardiac injury, thus were investigated in our model. *Nppa* was significantly increased in the MI‐SAT group versus SH‐SAT (with a main effect for MI) at 2 weeks, but at 4 weeks *Nppa* expression was significantly increased in MI groups as compared to respective (SH) controls (Table 4). At 8 weeks, *Nppa* gene expression was upregulated in the MI‐NC hearts only (Fig. 4). The transcriptional profile of structural proteins myosin heavy chain *α* (*Myh6*) and *β* (*Myh7*) were also evaluated as indicators of cardiac injury and stress (Table 4). Despite a trend of decreased expression, *Myh6* was not significantly altered at 2 or 4 weeks post‐MI with SAT. At 8 weeks, a significant depression was noted in *Myh6* expression for MI‐NC versus SH‐NC. *Myh7* expression was increased in the MI‐NC group at 2 weeks (with a main effect for MI) and at 4 weeks both MI groups exhibited higher transcript levels as compared to surgical controls (SH). Interestingly, by 8 weeks this expression pattern was abolished and instead, a main effect for diet was observed (Fig. 4). This time‐dependent profile of expression was also evident in the ratio of *Myh7*:*Myh6* (Table 4).

**Table 4.** Expression of cardiac injury and stress genes, *Nppa* and *Myh6/7*, in the injured heart with 2 or 4 weeks of post‐MI SAT.

	SH‐NC	SH‐SAT	MI‐NC	MI‐SAT
2 Weeks				
*Nppa*	0.07 ± 0.02	0.03 ± 0.01	0.20 ± 0.04*	0.24 ± 0.10**
*Myh6*	5.9 ± 1.1	5.8 ± 0.7	3.6 ± 0.6	5.6 ± 1.4
*Myh7*	9.9E‐5 ± 5.1E‐5	2.6E‐5 ± 0.3E‐5	21.4E‐5 ± 5.5E‐5*	19.4E‐5 ± 9.5E‐5**
*Myh 7*:*6*	16.4E‐6 ± 5.6E‐6	4.7E‐6 ± 0.6E‐6	64.6E‐6 ± 22.7E‐6**	40.9E‐6 ± 19.1E‐6*
4 Weeks				
*Nppa*	0.03 ± 0.02	0.02 ± 0.003	0.22 ± 0.003*	0.30 ± 0.1*
*Myh6*	6.1 ± 2.3	8.6 ± 2.4	6.0 ± 0.5	7.5 ± 1.0
*Myh7*	10.9E‐5 ± 4.6E‐5	6.6E‐5 ± 1.9E‐5	27.0E‐5 ± 4.9E‐5*	22.5E‐5 ± 5.9E‐5*
*Myh 7*:*6*	23.5E‐6 ± 11.1E‐6	9.5E‐6 ± 2.9E‐6	44.5E‐6 ± 6.3E‐5*	31.6E‐6 ± 8.4E‐6*

Relative gene expression values were determined using 18s rRNA for normalization and values are the mean ± SEM (*n* = 4–5).

* *P* < 0.05 versus surgical control (SH).

* A main effect (*P* < 0.05) for surgery (MI vs. SH).

### Alterations of metabolic regulatory and signaling proteins occur with MI and SAT

**Figure 5.**
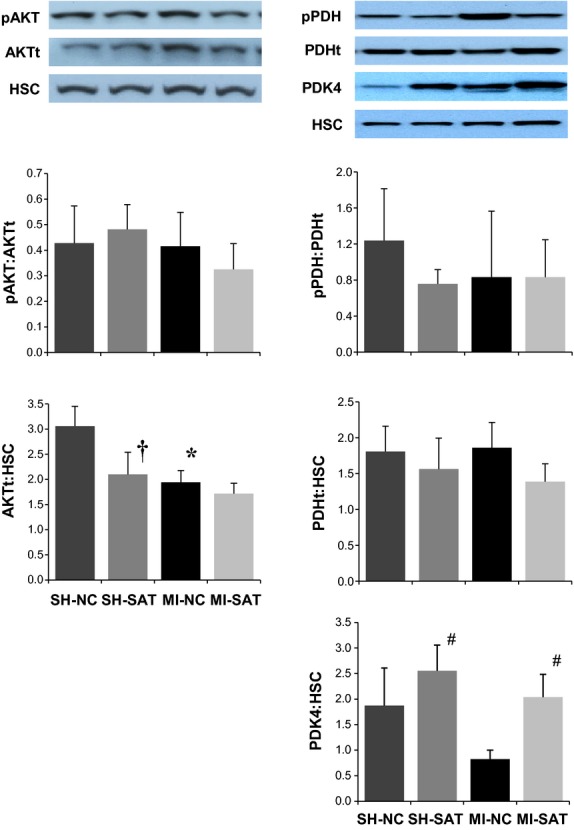
Protein abundance of phospho‐ and total AKT and PDH, along with PDK4 protein in post‐MI hearts with 2 weeks of high‐fat feeding. Representative blots are shown in addition to graphs of quantitative results from densiometric analysis of exposed films. Values shown are the mean ± SEM (*n *=**5–6). *P <**0.05 versus surgical control (SH). ^†^*P *<**0.05 versus dietary control (NC). ^#^A main effect (*P *<**0.05) for diet (SAT vs. NC).

As gene expression and functional changes were observed by 2 weeks post‐MI with SAT, several proteins related to metabolic phenotype and PI3K signaling were investigated in 2‐week LV samples. As shown in Figure 5 (left panel), phosphorylation of AKT (pAKT) was not altered by MI or SAT, although total AKT protein content was decreased in both the SH‐SAT and MI‐NC heart tissue as compared to the SH‐NC group. The abundance of PDK4 protein was increased in SH‐SAT hearts compared to SH‐NC, with a main effect for diet (Fig. 5, right panel). However, in spite of a diet‐induced increase in PDK4 protein, phospho‐PDH levels were not altered at 2 weeks in any of the groups. The total PDH protein pool was not affected.

## Discussion

### Summary of findings

This study further defines the cardioprotective effects of high dietary fat by identifying the impact on LV function conferred with high‐fat feeding following MI in a time‐dependent manner. The novel findings of our study include the demonstration of a cardioprotective effect of a high–saturated fat diet by 2 weeks which includes improvements in cardiac function/remodeling parameters and expression profiles of several genes and proteins related to energy metabolism along with markers of injury and stress in the heart. In terms of delineating the underlying mechanism(s) of dietary lipid‐induced cardioprotection, our findings support the notion that promoting FAO in the injured heart with endogenous substrates of metabolism is beneficial. This cardioprotection manifests early following injury by decreasing ventricular remodeling with improved function suggesting a shorter dietary intervention would confer benefit. Our work further hints at a primary role for the regulation of the metabolic phenotype in altering the evolution of LV dysfunction and has potential therapeutic value, although a dietary intervention to increase the caloric contribution of lipids in HF requires further investigation to fully appreciate how this would be implemented.

### High dietary fat alters the development of cardiac dysfunction acutely

Our previous work on the functional impact of high fat in the heart has shown that post‐MI, there is a notable improvement in contractility (±d*P*/d*t*) (Rennison et al. 2009; Berthiaume et al. 2010; Christopher et al. 2010; Cheng et al. 2011), without observable changes related to scar size or ventricular dimensions at 8 weeks between MI groups. In the current study, by 2 weeks of post‐MI SAT, a cardioprotective effect is evidenced by indications of diminished remodeling of the ventricle and improvements in contractile function. Systolic ventricular dimensions were smaller in the MI‐SAT group, with a trend toward improvement in +d*P*/d*t*, cardiac output, fractional shortening, ejection fraction, and strain. Other work documenting the progression of MI injury in the rat heart have shown that acutely, myocardial injury is initially noted as an expansion in ventricular volume which is then followed by a decline in contractility (Pfeffer et al. 1991; Litwin et al. 1994). This suggests a transition phase of injury in which the heart attempts to compensate for the loss in functional myocardium and that the ability of the remaining viable cardiomyocytes to meet the demands of pump function dictate the level of ventricular dysfunction. Our results suggest that lipids intervene in this compensatory phase to slow the progression of LV dysfunction. The short‐term effects of a high‐fat diet on cardiac function in the postinjury phase of MI represents an important observation as this duration of feeding limits the potential complications associated with increased peripheral adiposity and/or obesity making high fat dietary interventions potentially more suitable to clinical application.

### Gene and protein expression changes support a role for energy metabolism in lipid‐mediated cardioprotection post‐MI

The reduced ventricular remodeling reported with SAT is interesting in the context of the observed gene expression changes as it further supports a primary role for the regulation of metabolic phenotype in lipid‐induced cardioprotection after injury. Overall, the expression of energy metabolism‐related genes was depressed in the MI‐NC group. This could be interpreted as a global dampening of cellular function with MI, however, the structural (*myh7/6*, MHC*β*/*α*) and injury biomarker genes (*nppa*, ANP) were actually increased in expression. More likely, there is a targeted downregulation of energy metabolism in response to MI that occurs at 2 weeks (potentially earlier) that is countered by increased circulating lipids. Although *Acadm*, a FAO enzyme, expression was not altered, the other FA metabolism targets, *Acot1* and *Angptl4* assessed in this study were upregulated by SAT following MI, whereas normal chow‐fed rats undergoing MI demonstrated a trend of depression across these targets. The differences between expression in the MI groups across diet hints at a stimulation or at least preservation of the FA metabolism pathway. *Acot1* expression has been shown to be robustly and acutely induced by high‐fat feeding (Wilson et al. 2007) and may contribute to improved efficiency of FA handling within the cardiomyocytes. Additionally, Angptl4 may improve FA handling as well in its function as an extracellular lipase inhibitor as well as its angiogenic properties which have been shown to be integral to protection from ischemic injury in the heart (Galaup et al. 2012). Alterations in the cardiac metabolic phenotype post‐MI with SAT likely involves posttranslational modification of energy metabolism targets. Our data did not demonstrate an increase in pPDH to indicate decreased glucose oxidation with respect to diet as we have noted at 8 weeks (Berthiaume et al. 2012), however, the protein content of the kinase responsible for this inhibitory phosphorylation, PDK4, was elevated by SAT. Other work on increased dietary lipids has demonstrated additional sites on PDH that are more robustly phosphorylated acutely with high‐fat feeding and it may be that these sites would serve as better indicators of PDK4 activity in our model (Crewe et al. 2013). The notion of metabolic reprogramming acutely in cardiac injury is supported by other work on MI‐mediated cardiac dysfunction; 1 week subsequent to MI, there is a maximal peak in gene expression that is dominated by changes in energy metabolism targets (in contrast to the expression signature of physiological hypertrophy that does not impact FAO) (Beisvag et al. 2009). The observation of an early metabolic gene expression change is noteworthy as it is consistent with the metabolic shift classically associated with HF (Sack et al. 1996), but highlights that this defect in the metabolic program of the injured heart is evident prior to decompensation and not a decline precipitated by failure. Initiation of an altered metabolic program would likely be regulated by intracellular signaling pathways and the noted increase in *Pi3k* expression is an intriguing candidate as it regulates both metabolism and cell growth/proliferation (Kok et al. 2009). However, in our model we did not note a change to downstream targets of PI3K activity; AKT phosphorylation was not changed and the total protein pool was actually decreased. This may also be a time‐dependent observation or it may suggest alternative signaling cascades related to PI3K that have yet to be explored in the injured heart. Our data on a decrease in AKT protein are consistent with others' findings using a Western diet (high fat and carbohydrate) implying diet alone impacts this protein (Roberts et al. 2005) which is an axis of cellular signaling with pleiotropic effects on metabolism, cell growth, insulin signaling, cell death, etc. (Chaanine and Hajjar 2011). The signaling events accompanying SAT following cardiac injury deserve further investigation, but our data implies a unique, beneficial, regulatory program when SAT is introduced in the injured heart.

### High dietary fat improves cardiac markers of stress

We previously noted a diminution of the hypertrophic response at 8 weeks in the MI‐SAT group indicated by a smaller cardiomyocyte size and less induction of MHCβ protein content as compared to MI‐NC (Cheng et al. 2011). Here, we report a transcriptional shift from MHCα to β gene expression in MI‐NC (consistent with our findings at 8 weeks) that is diminished in MI‐SAT by 2 weeks supporting reduced mechanical stress with SAT. The time‐dependent effects noted in the gene expression data appear to be dynamic and may relate to other compensatory processes in the heart, consistent with the expanding notion of the overlap between metabolic and structural remodeling in cardiac injury (Doenst et al. 2013). How metabolic gene expression changes and pathway regulation overlaps with the evolution of the healing process has the potential to offer powerful insight into how transcriptional effector pathways converge in the heart and how regulation is achieved in terms of structural and metabolic remodeling and warrants further investigation.

### Limitations

There are a number of limitations to this study that likely contribute to some of the inconsistent trends across time in cardiac function. Unfortunately, no approach allows us to serially evaluate cardiac function by echocardiography and invasive hemodynamics in the same animal. This, coupled with the modest number of animals in each group (despite a large number of animals needed to complete the study), could contribute sufficient variability in our data to obscure differences in the MI groups that are more subtle than what we have reported in our previous studies following 8 weeks of SAT. Regardless, our data support an improvement in cardiac function with a high‐fat diet as dietary fat‐induced cardioprotection is evident at 2 weeks. Even with the opposing trend in volume remodeling at 4 weeks, improvements with SAT are evident by the increased stroke volume, cardiac output, and contractility. The cardioprotective effect of SAT at 2 and 4 weeks is also independently supported by the gene expression data which suggest the overall transcriptional repression of metabolism genes and MHC isoform switching noted in the MI‐NC hearts is diminished with SAT.

## Conclusions

Overall, this study provides additional evidence that a diet with a composition of high saturated fat introduced subsequent to cardiac injury is not detrimental to cardiac function in a rodent model of MI. Furthermore, this type of dietary intervention in the early stages of MI may prevent a maladaptive downregulation of energy metabolism that increases reliance on glucose over fatty acids (the primary substrate in the normal heart). These findings suggest that this cardioprotective effect, that we believe to be mediated through improvements in energy metabolism, is the product of cellular signaling that includes PI3K and is an upstream event in the progression of heart failure.

## Acknowledgments

A large debt of gratitude is owed to our friend and colleague William C. Stanley for his contributions to the study of cardiac metabolism along with his devotion to training and mentorship. The work of many, including the authors of this manuscript, has been informed and inspired by this unique individual. His untimely passing is a tragedy, both personally and professionally, for anyone who had the pleasure of knowing him; he will be sorely missed. The authors would also like to express our gratitude to Robert Harris and Pengfei Wu for kindly donating the PDK4 antibody, and Evan Rotar for his technical expertise with the Western blots.

## Conflict of Interest

None declared.

## Disclaimer

The research described in this article was completed while Margaret Chandler was an employee at Case Western Reserve University. The opinions expressed in this article are the authors' own and do not reflect the view of the National Institutes of Health, the Department of Health and Human Services, or the United States government.
